# Assessment of Heavy Metal Uptake in Potatoes Cultivated in a Typical Karst Landform, Weining County, China

**DOI:** 10.3390/foods11152379

**Published:** 2022-08-08

**Authors:** Xueqin Shi, Qiao Lin, Pengyu Deng, Tianyou Feng, Yuping Zhang

**Affiliations:** State Key Laboratory Breeding Base of Green Pesticide and Agricultural Bioengineering, Key Laboratory of Green Pesticide and Agricultural Bioengineering, Ministry of Education, Guizhou University, Guiyang 550025, China

**Keywords:** potatoes, soil, heavy metals, bioconcentration, correlation, pollution load index

## Abstract

The average content of heavy metals in Weining soil of karst landforms is generally higher than that of other agricultural regions. The aim of this study was to evaluate the heavy metal content in potatoes from Weining county and to analyze the correlation between the content of heavy metals in potatoes planted in the soil of karst landform and the soil’s environmental factors (soil heavy metals, soil pH, soil organic matter, altitude). Weining county (Guizhou province, China) is a typical karst landform, and has a potato production yield of 2.7 million tons. In this study, 56 soil and potato samples were collected from Weining county and the heavy metal content in the soils and potatoes was detected by inductively coupled plasma atomic mass spectrometry (ICP-MS). The content of Cr, Ni, and As in the soil was found to be higher, with almost half of the samples exceeding the maximum allowable levels. A total of 9 of the 56 samples tested had pollution load index values greater than 1.0, which indicates serious soil pollution. It was found that the ability of the potato to absorb heavy metals from the soil was very low, with the average bio-concentration factors of the metals Zn, Cu, Pb, Cr, Ni, and As being 0.087, 0.088, 0.0028, 0.0034, 0.0066, and less than 0.001, respectively. The content of the six heavy metals in the potatoes were all lower than the maximum permissible limit. The results show that a high As content in the soil could increase the content of Pb in potatoes, that a lower pH was beneficial to the bioaccumulation of Cr and Ni in potatoes, and that a high altitude is detrimental to the bioaccumulation of zinc and copper in potatoes. The HRI ranged between 1.12 × 10^−2^ and 5.92 × 10^−2^.

## 1. Introduction

With the rapid economic development occurring around the world, heavy metal pollution is common, and the area and degree of pollution is increasing, which poses a serious threat to the safety of agricultural production and human health [1,2]. Heavy metal pollution in crops has attracted significant attention as of late. The accumulation of heavy metals in soils is a major factor determining the high levels of heavy metals found in crops. The bioaccumulation of heavy metals—especially zinc (Zn), cuprum (Cu), lead (Pb), arsenic (As), chromium (Cr), nickel (Ni), and cadmium (Cd)—in the food chain presents a serious threat to human health [3,4]. For example, Pb poisoning can cause drowsiness, vomiting, irritability, loss of appetite, dizziness, and in severe cases, it can result in a coma or death [5]. Long-term exposure to Ni can also have serious health consequences, including rashes, fatigue, headaches, dizziness, respiratory diseases, decreased lung function, and even fatal cardiac arrest [6].

The potato is the most important non-grain food crop in the world. It is an important source of food owing to the energy, starch, vitamins, and minerals it contains [7]. Due to the growing demand for the food crop, Chinese potato production and exports to the international market are increasing year by year. Known as the ‘Hometown of the Southern China Potato’, Weining county ranks first in terms of the potato production in Guizhou province. At an elevation of nearly 2200 m, Weining county is the highest county in the province. It possesses a low average temperature and long hours of sunshine that make it highly suitable for potato farming. Weining county has a potato cultivation area of 1100 km^2^, with a total output of 2.7 million tons. The income earned from potato planting accounts for more than 20% of the average annual income of the county’s residents.

Located in western Guizhou province, Weining county is a typical karst landform composed of numerous types of limestone. It contains a large amount of Cd, As, Cu, Cr, and Zn in the carbonate rock and soil and is rich in mineral deposits [8,9]. According to the 2016 Bulletin on Geological Environment of Guizhou province, Guizhou had 4503 small-scale mines, including coal, phosphate, Fe, Zn, and Hg mines [10]. When comparing the content of heavy metals in different agricultural soils (Table S1), it is found that the average content of Cu, Cr, Zn, As, Pb, and Ni in Weining soils of karst landforms are generally higher than those in other regions [1,11,12,13,14,15,16]. In Weining county and the adjacent Hezhang county, many handmade Zn mining areas have existed since the 17th century. Zn smelting activities nearly ceased in 2004 due to concerns related to food safety and environmental pollution [17,18]. The vegetable uptake of metals is one of the major pathways through which soil metals enter into the food chain. Potatoes are in close contact with the soil, and they are buried in the soil for several months before ripening. Therefore, the content of Zn, Cu, Pb, Ni, and other heavy metals in potatoes has always been the focus of attention. Related studies have also reported the content of heavy metals such as Zn, Cu, Pb, and Ni in potatoes and soils [1,19,20,21].

However, Weining county, the primary production area for potatoes in China, has a karst landform that gives it different characteristics from the other major potato-producing areas in the world. Our group has reported that the concentrations of Cd were 0.41 to 10.0 mg/kg in the soil and were 0.023 to 0.18 mg/kg in the potatoes in Weining county. A regression model to predict the concentration of cadmium in the potatoes based on soil properties was developed in Weining county [22]. To date, no analysis has been performed regarding the heavy metal content (such as Cu, Cr, Zn, As, Pb, and Ni) of potatoes from Weining county. The correlation between the presence of heavy metals (such as Cu, Cr, Zn, As, Pb, and Ni) in potatoes and the environmental factors present in the karst landforms has not been investigated.

In this study, we analyzed and tested 56 samples from 15 towns and determined the Cu, Cr, Zn, As, Pb, and Ni concentrations in soils and potatoes in Weining county. The correlation between the concentrations of these heavy metals in potatoes and the environmental factors was analyzed. The bioconcentration factor (BCF), pollution load index (PLI), daily intake of metal (DIM), and health risk index (HRI) were obtained. Further safety assessments were carried out based on the daily consumption of potatoes cultivated in karst landforms.

## 2. Materials and Methods

### 2.1. Study Area

**Figure 1:**
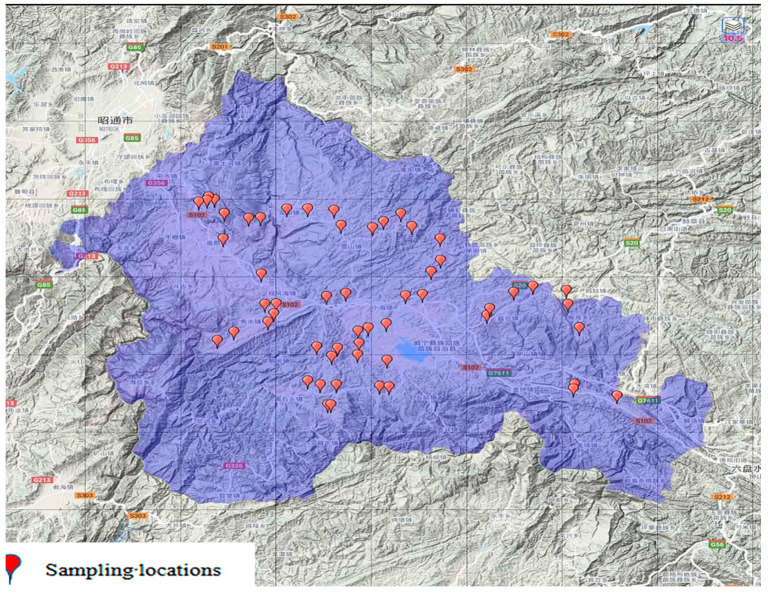
A sampling map of 56 potatoes and soil.

Weining county, located in the western part of Guizhou province (103°36′–104°45′ E, 26°36′–27°26′ N), has a total area of 6295 km^2^. The potato and soil samples were collected from the same 25 m^2^ open field area. A total of 56 soil samples and 56 potato samples were collected from 15 towns in the county. A sampling map of 56 potatoes and soil is shown in Figure 1.

### 2.2. Collection of Samples

Approximately 2 kg of soil samples were selected from 7 locations in the vegetable field (0 to 25 cm). The soil samples were mixed and homogenized, placed in polyethene bags, and sent to the laboratory. Air-dried soil samples were passed through a 2 mm nylon mesh sieve.

Approximately 2 kg of potato samples were randomly selected at 7 corresponding soil locations and taken to the laboratory. After washing with distilled water, the potatoes were chopped, repeatedly sampled using a quarter method, dried in an oven at 65 °C for 4 days, and weighed before and after drying to calculate their moisture content. The moisture content of each potato sample was shown in Table S2 in the supporting information.

### 2.3. Sample Processing

A total of 0.1 g (accurate to 0.0001 g) of soil sample was placed into a digester and mixed with 2 mL of concentrated nitric acid and 1 mL of hydrofluoric acid [23]. Next, the digester was covered, placed in an oven for digestion at 180 °C for 8 h and cooled overnight. The acid was evaporated away by placing the digester on a 180 °C plate until it was nearly dry. Then, it was cooled to room temperature and the residue in the digester was washed with 2% HNO_3_ and transferred into a 25 mL volumetric flask. The digested solution was analyzed using inductively coupled plasma atomic mass spectrometry (ICP-MS). Each sample was analyzed in triplicate. The method was verified using soil material standards (GSS-14 and GSS-4). The relative standard deviation of the parallel determinations of heavy metal concentrations in the soil samples was less than 15%.

A total of 0.5 g (accurate to 0.0001 g) of potato sample was placed into a 50 mL beaker, mixed with 10 mL of nitric acid, covered with a porcelain lid, and allowed to soak overnight. The next day, 2 mL of perchloric acid was added. Digestion continued on a hot plate at 180 °C until the solution became colorless [24]. Afterwards, the lid was removed, and the digestion solution was evaporated to near dryness at 140 °C. The residue was dissolved in 2% HNO_3_ and transferred to a 25 mL volumetric flask. The concentration of Zn, Cu, Pb, As, Cr, and Ni in digestive juice was determined using ICP-MS. Each sample was analyzed in triplicate. The national standard carrot sample GSB-25 and tangerine sample GSB-11 were used to control the accuracy of the method. The relative standard deviations for the determination of the parallel of heavy metal concentrations in the potatoes were all less than 15%.

## 3. Data Analysis

All the data were analyzed using SPSS 17.0. The K-means clustering algorithm analysis of the content of each metal was used to identify groups, then the significance analysis was evaluated between groups at *p* < 0.05. The Pearson correlation test was used to analyze the correlation between the content of heavy metals in the potatoes and soil organic matter (OM), soil pH, and elevation.

Other parameters, such as the BCF, PLI, DIM, and HRI, were also determined.

This section may be divided by subheadings. It should provide a concise and precise description of the experimental results, their interpretation, as well as the experimental conclusions that can be drawn.

### 3.1. Analysis of Soil pH and OM

The soil and water were mixed at 1:2.5 and the pH was measured with a Sartorius pH meter. Deoxidation was performed with K_2_Cr_2_O_7_ and then the titration method was used to determine the content of organic matter in the soil [25]. Each sample was made in three parallels during the process. In this study, the soil pH varied from 4.36~8.41, with an average value of 5.74. The amount of organic matter (OM) varied from 12.3~102 g/kg with an average value of 50.7 g/kg, as shown in Table 1.

### 3.2. Bioconcentration Factor

To study the transfer of metals from the soil to the potatoes, the BCF values were calculated as follows [4,26].

BCF = C_potato_/C_soil_ (1)

C_potato_ and C_soil_ represent the concentrations of heavy metals in the potato and soil (dry weight), respectively.

### 3.3. Pollution Load Index of the Soil

The extent of pollution by trace metals was assessed by employing the method based on PLI developed by Tomlinson et al. [27], expressed by the following equations:

Contamination Factor (CF) = C_soil_/C_PML_ (2)

PLI = (CF_1_ × CF_2_ × CF_3_……CF_n_)^1/n^ (3)

where C_soil_ represents the concentration of heavy metal in soil and C_PML_ represents the permissible maximum limit set by the World Health Organization for heavy metals in the soil. CF = contamination factor and n = number of metals [28].

### 3.4. Daily Intake of Metals from Potatoes and the Associated Health Risk Index

A health risk assessment for consumers based on their intake of metal-contaminated crops was characterized using an HRI. The HRI value was calculated using the formula below [29,30,31].

HRI = DIM/R_f_D (4)

where DIM (mg/kg/day) is the daily intake of heavy metals via the exposure pathway through the ingestion of vegetables and R_f_D is the reference dose. The DIM value was calculated using the following formula [32,33].

DIM = C_metal_ × D_food intake_/B_average weight_ (5)

C_metal_ represents the concentration of heavy metals in the potato (fresh weight), D_food intake_ represents the average ingestion of potatoes per day (0.128 kg) in 2014 [6], and the B_average weight_ represents the average body weight for adults, equal to 60 kg [34]. The R_f_D standard values recommended by the ‘Integrated Risk Information System’ for Zn, Cu, As, and Ni are 0.37, 0.04, 3 × 10^−4^, and 0.02 mg kg^−1^ day^−1^, respectively [35]. The R_f_D value for Pb was 0.0035 mg kg^−1^ day^−1^ [36] and Cr was 1.38 × 10^−2^ mg kg^−1^ day^−1^ [6].

## 4. Results

### 4.1. Assessment of Heavy Metals in the Soil Sample

The concentrations of heavy metals Zn, Cu, Pb, As, Cr, and Ni in soil are shown in Table 2 and Table S3 (in Supporting Information). The average concentrations of Zn, Cu, Pb, As, Cr, and Ni in the soils were 175, 47.8, 59.3, 25.8, 119, and 50.8 mg/kg. Zhang et al. [22] has reported that the average concentration of cadmium in Weining potato soil was 2.60 mg/kg. So, the concentration order of heavy metals in the potato cultivation soil of Weining county was Zn > Cr > Pb > Ni > Cu > As > Cd. The results of the cluster analysis show that when the number of the cluster was five, the difference between the various groups reached a significant level (*p* < 0.05). The maximum allowable levels of metal Zn, Cu, Pb, As, Cr, and Ni in the soil are as follows: 300, 100, 100, 20, 100, and 50 mg/kg, respectively [37]. In the 56 soil samples, the Zn content of 5 samples, Cu content of 2 samples, and Pb content of 3 samples were found to exceed the standard limit. However, the As content of 39 samples, Cr content of 35 samples, and the concentration of Ni in 26 samples were higher than the standard limit.

(abcde: Different lowercase letters in the same column indicate significant differences between groups, *p* < 0.05.).

**Figure 2:**
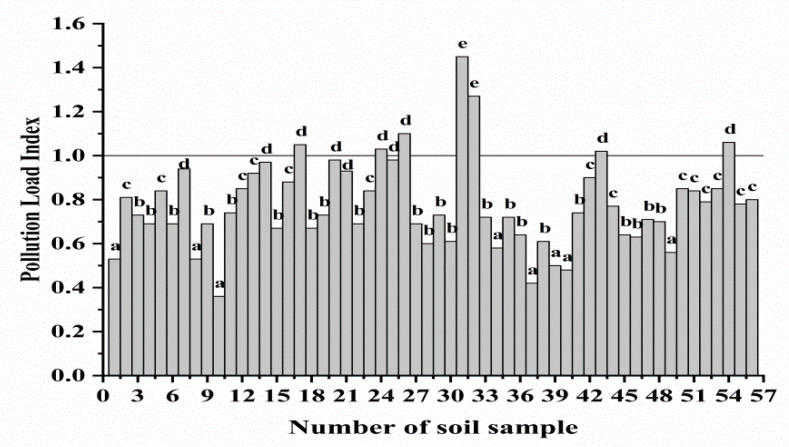
Pollution load index (PLI) distribution of 56 locations’ soil. (abcde: Different lowercase letters indicate significant differences between groups, *p* < 0.05.)

The degree of soil metal contamination was determined by the comprehensive PLI. The pollution index was calculated based on the contamination factor of each metal [27]. If the PLI value of the metal in the soil is greater than 1, the soil is considered to be contaminated, while uncontaminated soil samples have PLI values less than 1 [38]. The PLI values of the 56 samples are shown in Figure 2 and Table S4 (in Supporting Information). The PLI ranged from 0.357 to 1.56. Nine of fifty-six samples in the Weining region had a PLI value greater than 1.0. The PLI values of soil samples #26, #31, and #32 exceeded 1.3. The Cr, Ni, and As content in the three soils all exceeded the limit values. The Zn content of soil samples #31 and #32 were also very high, with a Zn concentration of 371.8 and 468.6 mg/kg, respectively. (Table S3 in Supporting Information). The results of the cluster analysis show that when the number of cluster was five, the difference between the various groups reached a significant level (*p* < 0.05).

### 4.2. Assessment of Heavy Metals in the Potato Sample

The results of the heavy metal content in the potato samples are summarized in Table 3. The average concentrations of Zn, Cu, Pb, Cr, and Ni in the potatoes were 2.73, 0.675, 0.027, 0.072, and 0.055 mg/kg (fresh weight), respectively. The concentrations of As in all the potato samples were below the detection limit (0.01 mg/kg). The concentrations of each heavy metals in the potatoes were all below the permissible maximum limit [39]. Zhang et al. [22] reported that the average concentration of Cd in potatoes in Weining county is 0.083 mg/kg (fresh weight). The sequence of metal concentrations in the potatoes was Zn > Cu > Cd > Cr > Ni > Pb > As. The results of the cluster analysis show that when the number of the cluster was five, the difference between the various groups reached a significant level (*p* < 0.05).

The BCF reflects the plant’s ability to accumulate trace elements. Table 4 listed the BCF values for each metal element in potatoes. The average BCF of Zn, Cu, Pb, Cr, and Ni in the potatoes were 0.107, 0.088, 0.0028, 0.0034, and 0.0066, respectively. The average BCF for As was less than 0.001. The order of the BCFs of the metals (Zn, Cu, Pb, As, Cr, and Ni) in Weining County was Zn > Cu > Ni > Cr~Pb > As. The results of the cluster analysis show that when the number of the cluster was five, the difference between the various groups reached a significant level (*p* < 0.05).

### 4.3. Correlation between Heavy Metal Concentrations in Potatoes and the Environmental Parameters

In this study, the soil pH varied from 4.36–8.41, with an average value of 5.74. The amount of OM varied from 12.3–102 g/kg and the OM average value was 50.7 g/kg. The elevation varied from 2151–2744 m with an average value of 2359 m, as shown in Table 1.

The results of the correlation analysis (Pearson Correlation, 2-tailed) are shown in Table 5. It can be concluded that the content of As in the soil affects the content of Pb in the potatoes (Table 3), and there was a significant correlation between them (correlation coefficient = 0.301, *p* < 0.05). However, there was no correlation between the other metals in the soil and in the potatoes. There was a negative correlation between the pH of the soil and the Ni content (correlation coefficient = −0.591, *p* < 0.01) and Cr content (correlation coefficient = −0.344, *p* < 0.01) in the potatoes. There was no significant correlation between the OM content and the heavy metal content in the potatoes. Elevation affected the content of Zn (correlation coefficient = −0.292, *p* < 0.05) and Cu (correlation coefficient = −0.444, *p* < 0.01) in the potatoes.

### 4.4. Health Risk Index and Daily Intake of Metals of Potatoes

Potatoes are a very important global food crop. To measure the risk exposure to consumers, the HRI was used. First, the DIM was calculated using Equation (5). Many researchers have used the DIM value to assess human daily metal intake and its associated harm [40,41,42]. The DIM values in this study are shown in Table 6; the order of the DIM values was Zn > Cu > Cr > Ni > Pb > As. The DIM values of these metals did not exceed 5.81 × 10^−3^ mg/kg.

The reference values of the limits of daily heavy metal intake were obtained using the HRI. Then, the HRI was calculated using Equation (4) and is shown in Table 6. In this study, the values of the HRI for the six heavy metals varied from 0.0112 to 0.0592. The observed order was Ni > Cu > Pb > Zn > Cr > As (Table 6). There is no obvious risk to human health if the HRI is <1, but a risk is present if the HRI is >1 [29,32]. The HRI value indicates that the daily consumption of potatoes cultivated in Weining karst landforms does not pose a hazard to human health.

## 5. Discussion

The concentration order of various heavy metals in soils varies from place-to-place owing to the different soil backgrounds and pollution conditions. Golia et al. [1] has reported the order of concentrations of heavy metals in the soil of locally planted potatoes in Trikala was Zn (9.87 mg/kg) > Cu (0.925 mg/kg) > Pb (0.051 mg/kg) > Ni (0.024 mg/kg) > Cr (0.018 mg/kg) > Cd (0.015 mg/kg). Mehrdad et al. [13] has reported that the order of concentrations of heavy metals in the soil of locally planted potatoes in the Hamadan province, western Iran, was Zn (55.6 mg/kg) > Cr (40.1 mg/kg) > Ni (32.9 mg/kg) > Cu (26.8 mg/kg) > Pb (10.4 mg/kg) > As (8.60 mg/kg) > Cd (2.20 mg/kg). In this study, the concentration order of the heavy metals in the potato cultivation soil was Zn (175 mg/kg) > Cr (119 mg/kg) > Pb (59.3 mg/kg) > Ni (50.8 mg/kg) > Cu (47.8 mg/kg) > As (25.8 mg/kg) > Cd (2.60 mg/kg) [22]. It can be concluded that the content of heavy metals in the soil in the karst potato growing areas is higher than that in other potato growing areas. Since the 17th century, Zn smelting activities using indigenous methods have been carried out in Weining county [18], which may be the reason for the excessive Zn content in some places. Currently, Weining is a major agricultural production county. The research has found that the pollution of Cr, Ni, and As in the soil was more serious than previously believed.

Golia et al. [1] reported the concentration levels of heavy metals in potatoes (fresh weight) in the Trikala region: Zn (0.201 mg/kg) > Cu (0.101 mg/kg) > Cr (0.089 mg/kg) > Pb (0.077 mg/kg) > Ni (0.057 mg/kg) > Cd (0.008 mg/kg). Šrek [19] reported the concentrations of heavy metals in potatoes (dry weight) in the Ruzyne region to be in the following order: Zn (16.1 mg/kg) > Cu (4.30 mg/kg) > Pb (0.60 mg/kg) > Ni (0.58 mg/kg) > Cr (0.19 mg/kg) > As (0.06 mg/kg) > Cd (0.04 mg/kg). Gebrekidan et al. [20] reported that the concentrations of heavy metals in potatoes (dry weight) in the Tahtay region was Pb (2.58 mg/kg) > Cu (2.52 mg/kg) > Zn (1.40 mg/kg) > Cr (0.39 mg/kg) > Ni (0.25 mg/kg) > Cd (0.18 mg/kg). The average concentrations of heavy metals in Weining potatoes (fresh weight) were Zn (2.73 mg/kg) > Cu (0.675 mg/kg) > Cr (0.072 mg/kg) > Ni (0.055 mg/kg) > Pb (0.027 mg/kg) > As (<0.01 mg/kg), in our study. Evidently, the content levels of heavy metals in potatoes varied widely across the regions. The order of the levels of different heavy metals in potatoes is not the same in different agriculture soils.

Khan et al. [21] reported the order of the BCF of the heavy metals (Mo, As, Se, Fe, Cu, Zn, Ni, and Pb) in potatoes irrigated by underground water in the Punjab region of Pakistan region as Zn (20.2) > Cu (4.63) > Mo (1.27) > Ni (1.09) > Fe (1.05) > Se (0.35) > Pb (0.19) >As (0.06). Zhang et al. [22] reported that the average BCF of the heavy metal Cd in Weining potatoes was 0.24. The order of the BCFs of the metals (Zn, Cu, Pb, As, Cr, Cd, and Ni) in Weining County was Cd (0.24) > Zn (0.107) > Cu (0.088) > Ni (0.007 > Cr (0.003) ≈ Pb (0.003) > As (<0.001). In both studies, the ability of potatoes to absorb heavy metals from soil was found to be Zn > Cu > Ni > Pb > As; however, the ability of potatoes to absorb heavy metals was much larger in soils in central Punjab, Pakistan. Mehrdad et al. [13] reported the order of the BCF of the heavy metals (As, Ni, Cd, Cu, Pb, Cr, and Zn) in potatoes from soils that overused phosphate fertilizers in western Iran to be As (0.46) > Ni (0.41) > Cd (0.40) ≈ Cu (0.40) ≈ Pb (0.40) > Cr (0.39) ≈ Zn (0.39), which seemed to suggest to suggest that due to the effect of phosphate fertilizer, the BCF of the potato to various heavy metals tends to be the same (about 0.4). It can be concluded that there were also differences in the BCFs of metals in different conditions. Therefore, it was necessary to study the concentration and bioconcentration of heavy metals in potatoes cultivated in karst landforms.

The content of heavy metals in plants has been proven to be influenced by many factors, such as the content of heavy metals in the soil [4,43], soil pH [4,5,44], the amount of OM in the soil [4,22,45], and elevation [22]. Through the correlation analysis, it can be concluded that the content of metals found in potatoes cultivated in karst field environments of Weining were affected by many factors. In Weining, the high As content in the soil could increase the content of Pb in potatoes, the lower pH was beneficial to the bioaccumulation of Cr and Ni in potatoes, and the high altitude was detrimental to the bioaccumulation of zinc and copper in the potatoes.

## 6. Conclusions

Through an analysis of six heavy metals (Zn, Cu, Pb, As, Cr, and Ni) in the soil and potato samples, it can be concluded that the heavy metal pollution in Weining soil with a typical karst landform was caused by As, Cr, and Ni. The ability of the potato to absorb heavy metals from the soil was very small, so the concentration of heavy metals in potatoes was below the maximum threshold. There was a significant positive correlation between the content of As in the soil and the content of Pb in potatoes. The content of As in 70% of the soil samples exceeded the standard, which has a potential impact on the bioaccumulation of Pb in Weining potatoes. In this study, a lower pH was more favorable for the transfer of Ni and Cr to potatoes, and a high elevation was not a conducive factor for the bioaccumulation of Zn and Cu in the potatoes. The bioaccumulation of heavy metals (Zn, Cu, Pb, As, Cr, and Ni) in the potatoes and the HRI obtained by the DIM in this study were very low, further indicating that there is no potential health risk in the consumption of potatoes from the Weining karst landforms.

## Figures and Tables

**Table 1:** The environmental parameters and moisture content in potatoes.

Contents	pH	OM	Elevation (m)	Moisture Content (%)
Range	4.36–8.41	12.3–101.9	2151–2744	0.78–0.85
Mean	5.74	50.7	2360	0.81
Median	5.45	48.35	2332	0.81

**Table 2:** Analysis of variance for metals concentrations (mg/kg) in soil (*n* = 56).

Metals			Zn	Cu	Pb	As	Cr	Ni
Mean ± SD			181 ± 81.7	47.8 ± 22.0	59.3 ± 20.9	25.8 ± 10.1	119 ± 41.2	50.8 ± 15.6
Maximum value			469	131	115	62.8	292	97.2
Minimum value			62.8	18.9	20.0	9.74	62.1	24.9
Median			163	41.4	52.7	23.5	108	47.6
Group	Ⅰ	Number	16	7	3	17	22	4
		Range	62.8–131 ^a^	18.9–26.4 ^a^	20.0–29.2 ^a^	9.74–19.3 ^a^	62.1–100 ^a^	24.9–29.65 ^a^
	Ⅱ	Number	25	30	36	22	24	25
		Range	137–202 ^b^	32.8–45.9 ^b^	36.3–68.9 ^b^	20.3–29.0 ^b^	104–135 ^b^	32.0–47.7 ^b^
	Ⅲ	Number	10	10	8	14	6	18
		Range	211–292 ^c^	49.3–64.6 ^c^	72.2–77.8 ^c^	30.9–41.0 ^c^	145–183 ^c^	49.8–63.4 ^c^
	Ⅳ	Number	4	7	6	2	3	7
		Range	334–377 ^d^	67.2–88.5 ^d^	80.5–95.2 ^d^	44.5–49.5 ^d^	198.9–225 ^d^	68.0–77.8 ^d^
	Ⅴ	Number	1	2	3	1	1	2
		Range	469 ^e^	131–131 ^e^	104–115 ^e^	62.8 ^e^	292 ^e^	93.1–97.2 ^e^
Exceeding the maximum permissible sample size			5	2	3	39	35	26
Permissible Maximum limit (mg/kg)			300	100	100	20.0	100	50.0

(abcde: Different lowercase letters in the same column indicate significant differences between groups, *p* < 0.05.).

**Table 3:** Analysis of variance for metals concentrations (mg/kg) in potatoes (fresh weight *n* = 56).

Metals			Zn	Cu	Pb	As *	Cr	Ni
Mean ± SD			2.73 ± 1.02	0.675 ± 0.228	0.027 ± 0.018	<0.010	0.072 ± 0.153	0.055 ± 0.032
Maximum value			7.42	1.18	0.095	<0.010	0.659	0.202
Minimum value			1.56	0.232	0.010	<0.010	0.010	0.018
Median			2.48	0.660	0.024	<0.010	0.023	0.047
Group	Ⅰ	Number	20	5	16	--	46	17
		Range	1.56–2.22 ^a^	0.232–0.327 ^a^	0.010–0.017 ^a^	--	0.010–0.036 ^a^	0.018–0.036 ^a^
	Ⅱ	Number	21	15	19	--	5	17
		Range	2.35–3.02 ^b^	0.397–0.561 ^b^	0.018–0.025 ^b^	--	0.042–0.107 ^b^	0.039–0.052 ^b^
	Ⅲ	Number	12	14	13	--	1	12
		Range	3.07–4.20 ^c^	0.574–0.71 ^c^	0.029–0.038 ^c^	--	0.492 ^c^	0.059–0.079 ^c^
	Ⅳ	Number	2	12	6	--	3	9
		Range	5.04–5.15 ^d^	0.744–0.907 ^d^	0.043–0.054 ^d^	--	0.529–0.543 ^d^	0.086–0.109 ^d^
	Ⅴ	Number	1	10	2	--	1	1
		Range	7.42 ^e^	0.964–1.18 ^e^	0.094–0.095 ^e^	--	0.659 ^e^	0.202 ^e^
Exceeding the maximum permissible sample size			0	0	0	0	0	0
Permissible maximum limit (mg/kg)			60	40	0.3	7	2.3	67.90

(* below the test line 0.01 mg/kg; abcde: Different lowercase letters in the same column indicate significant differences between groups, *p* < 0.05.).

**Table 4:** Bio-concentration factor for potato (dry weight)/soil system.

Contents			Zn	Cu	Pb	As	Cr	Ni
Range			0.010–226	0.024–0.207	<0.001–0.009	<0.001	<0.001–0.032	0.001–0.024
Median			0.071	0.075	0.002	<0.001	0.001	0.005
Mean			0.087	0.088	0.003	<0.001	0.003	0.007
Group	Ⅰ	Number	9	10	8	--	2	21
		Range	0.010–0.045 ^a^	0.024–0.046 ^a^	<0.001 ^a^	--	<0.001 ^a^	0.001–0.004 ^a^
	Ⅱ	Number	26	22	23	--	48	26
		Range	0.052–0.082 ^b^	0.049–0.085 ^b^	0.001–0.002 ^b^	--	0.001–0.004 ^b^	0.005–0.009 ^b^
	Ⅲ	Number	12	15	20	--	2	5
		Range	0.088–0.128 ^c^	0.097–0.125 ^c^	0.003–0.005 ^c^	--	0.007–0.016 ^c^	0.011–0.014 ^c^
	Ⅳ	Number	5	6	4	--	2	3
		Range	0.143–0.171 ^d^	0.139–0.173 ^d^	0.006–0.008 ^d^	--	0.022–0.023 ^d^	0.017–0.020 ^d^
	Ⅴ	Number	4	3	1	--	2	1
		Range	0.192–0.226 ^e^	0.193–0.207 ^e^	0.009 ^e^	--	0.028–0.032 ^e^	0.024 ^e^

(abcde: Different lowercase letters in the same column indicate significant differences between groups, *p* < 0.05.).

**Table 5:** Pearson correlation between the heavy metal content in potatoes and the environmental parameters.

Environment	Heavy Metals in Potatoes (mg kg^−1^)					
Parameters	Zn	Cu	Pb	As	Cr	Ni
[Zn] _soil_	0.220	0.055	0.231	--	0.155	−0.048
[Cu] _soil_	−0.082	−0.031	−0.070	--	0.213	−0.127
[Pb] _soil_	0.212	−0.163	0.109	--	−0.097	−0.255
[As] _soil_	0.105	0.085	0.301 *	--	0.182	−0.088
[Cr] _soil_	−0.033	−0.134	0.142	--	0.083	−0.076
[Ni] _soil_	0.070	0.002	0.108	--	0.039	−0.071
pH	−0.208	−0.095	−0.028	--	−0.344 **	−0.591 **
OM	0.208	−0.117	−0.037	--	−0.135	−0.397
Elevation	−0.292 *	−0.444 **	0.250	--	0.034	−0.066

** = Significant at 0.01 levels; * = Significant at 0.05 levels

**Table 6:** Health risk index (HRI) and daily intake of metals (DIM mg/kg/day).

Plant	Hazard Quotient						
Potato	Metals	Zn	Cu	Pb	As	Cr	Ni
	DIM	5.81 × 10^−3^	1.44 × 10^−3^	5.79 × 10^−5^	<2.1 × 10^−5^	1.54 × 10^−4^	1.18 × 10^−4^
	R_f_D (mg kg^−1^ day^−1^)	0.37	0.04	3.5 × 10^−3^	3 × 10^−4^	1.38 × 10^−2^	0.02
	HRI	1.57 × 10^−2^	3.59 × 10^−2^	1.65 × 10^−2^	--	1.12 × 10^−2^	5.92 × 10^−2^

## Data Availability

The data that support the findings of this study are available from the corresponding author upon reasonable request.

## Supplementary Materials

The following supporting information can be downloaded at: https://www.mdpi.com/article/10.3390/foods11152379/s1, Table S1: Heavy metal content in soil in different agricultural regions (mg kg-1); Table S2: The environment parameters and moisture content in potato; Table S3: Heavy metal concentrations in 56 soil samples (mg/kg); Table S4: Pollution load index for metals in soil; Table S5: Heavy metal concentration in 56 potato samples (wet weight mg/kg). Table S6: Bio-concentration factor potato (dry weight)/soil system.
