# Supplementary material for: Assessment of Heavy Metal Uptake in Potatoes Cultivated in a Typical Karst Landform, Weining County, China
Source: Foods. 2022 Aug 8;11(15):2379. doi: 10.3390/foods11152379 (PMC9367836; doi:10.3390/foods11152379)
Supplement: Supplementary file 1 [file foods-11-02379-s001.zip › foods-1835333-supplementary.pdf]

SUPPORTTING INFORMATION

Assessment of heavy metal uptake in potato cultivated in a typical karst landform, Weining county, China

Xueqin Shi [1], Qiao Lin[1], Pengyu Deng[1], Tianyou Feng [1], Yuping Zhang[1]*

[1] State Key Laboratory Breeding Base of Green Pesticide and Agricultural Bioengineering, Key Laboratory of Green Pesticide and Agricultural Bioengineering, Ministry of Education, Guizhou University, Guiyang 550025, P. R. China.

* Correspondence: zhangyupinggz@163.com; Tel.: (+86)851 88292090

Table S1: Heavy metal content in soil in different agricultural regions (mg kg^{-1})

Region	Metals	Cu	Cr	Zn	As	Pb	Ni	References
Weining, Guizhou, China	Mean	47.8	119	175.01	25.8	59.3	50.8	In this study
	Range	18.92-131.35	62.05-292.04	22.94-468.55	9.74-62.83	19.96-115.46	24.89-97.15	
Trikala, Greece	Mean	0.925	0.018	9.87	-	0.051	0.024	Golia et al, 2008[1]
	Range	-	-	-	-	-	-	
Netherlands	Mean	-	-	-	12	31	-	Wiersma et al, 1986[11]
	Range	-	-	-	0.1-110	0-460	-	
Yangzhong District, Jiangsu, China	Mean	32.1	80.9	79.4	12.0	26.7	33.4	Huang et al, 2007[12]
	Range	24.9-40.2	74.1-90.4	62.8-101	9.2-14.3	19.9-35.7	29.3-45.1	
Hamadan, Iran	Mean	26.7	40.1	55.6	8.6	10.4	32.9	Mehrdad et al, 2013[13]
	Range	1.46-50.11	18.2-53.8	0.64-139.4	2.4-17.03	0.55-30.1	0.35-54.9	
Four regions in Zhejiang, China	Mean	-	-	-	-	-		Wang et al, 2020[14]
	Range	27.0-83.4	47.1-91.2	120.8-359.0	6.44-17.64	32.6-216.4	19.0-48.7	
Guigang, guangxi, China	Mean	24.45	61.12	66.42	20.31	22.67	44.14	Shan et al, 2020[15]
	Range	4.89-52.11	6.91-105.12	27.87-122.80	3.11-39.43	0.45-61.17	13.55-98.44	
Turpan Basin, China	Mean	-	59.00	-	9.57	13.41	21.60	Marhaba et al, 2021[16]
	Range	-	47.30-74.10	-	6.86-13.41	8.34-25.40	16.10-28.50	

[1] Golia, E. E.; Dimirkou, A.; Mitsios, I. K. Influence of some soil parameters on heavy metals accumulation by vegetables grown in agricultural soils of different soil orders. *Bull Environ Contam Toxicol*. **2008**, *81(1)*, 80-84.

[11] Wiersma, D.; Berend, J.; Nicolaas, G. Cadmium, lead, mercury, and arsenic concentrations in crops and corresponding soils in the Netherlands. *J Agric Food Chem*. **1986**, *34*, 1067-1074.

[12] Huang, S. S.; Liao, Q. L.; Hua, M.; Wu, X. M.; Bi, K. S.; Yan, C. Y.; Chen, B.; Zhang, X. Y. Survey of heavy metal pollution and assessment of agricultural soil in Yangzhong district, Jiangsu province, China. *Chemosphere*. **2007**, 67(11), 2148-2155.

[13] Mehrdad, L. Bahareh, M. Hajar et al.Heavy metal risk assessment for potatoes grown in overused phosphate-fertilized soils. *Environ Monit Assess*. **2013**, *185(2)*,

1825-1831.

[14] Wang, Y. L.; Ji, T. W.; Yu, D. H.; Xiao, M. Investigation on heavy metal contents in soils and vegetables of some vegetable bases in zhejiang province. *J Zhejiang Agric Sci*. **2020**, 61(07), 1310-1312.

[15] Shan, Z. Q.; Shi, S. M.; Ma, R. K.; Yang, X. L. Soil heavy metals pollution and ecological risk assessment in citrus production areas of Guigang, Guangxi province. *Sichuan Environ*. **2020**, *39(04)*, 136-141.

[16] Marhaba, T.; Mamattursun, E.; Wang, W. W. Heavy metal contamination and potential ecological risk of vineyard soil in Turpan Basin. *Environ Monit Chin*. **2021**, *37(01)*, 112-119.

Table S2: The environment parameters and moisture content in potato

Sample no	pH	OM	Elevation (m)	Moisture content (%)
1	5.45	23.1	2196	0.806
2	6.68	43.3	2199	0.808
3	7.29	43.5	2172	0.815
4	5.71	30.1	2187	0.838
5	5.26	71.3	2324	0.806
6	5.74	75.9	2546	0.810
7	5.22	57.8	2728	0.819
8	5.55	82.1	2608	0.828
9	7.34	20.8	2379	0.826
10	4.9	12.3	2474	0.805
11	5.38	48.5	2534	0.840
12	5.94	63.2	2314	0.805
13	6.33	78.0	2404	0.816
14	5.97	47.9	2377	0.807
15	5.90	68.2	2312	0.794
16	5.42	29.2	2195	0.810
17	6.36	61.8	2324	0.795
18	4.88	64.8	2291	0.783
19	4.90	53.6	2336	0.813
20	6.60	76.5	2313	0.801
21	5.45	68.4	2301	0.790
22	5.05	86.0	2437	0.807
23	6.54	56.6	2456	0.813
24	4.36	82.7	2383	0.838
25	7.95	52.0	2360	0.796
26	5.93	68.3	2331	0.805
27	8.22	23.0	2174	0.800
28	5.32	45.6	2182	0.803
29	7.31	50.7	2226	0.806
30	4.75	49.2	2151	0.785
31	4.59	39.9	2240	0.817
32	4.56	77.8	2255	0.820
33	5.95	75.8	2352	0.821
34	5.00	59.3	2410	0.832
35	5.58	86.1	2534	0.850
36	5.44	101.9	2520	0.822
37	4.64	25.0	2332	0.806
38	4.64	39.5	2180	0.805
39	4.58	34.1	2184	0.818
40	5.12	42.1	2325	0.813

41	6.10	30.7	2583	0.818
42	5.49	61.3	2744	0.817
43	5.40	28.8	2658	0.802
44	5.50	38.1	2572	0.818
45	8.41	29.2	2371	0.790
46	5.12	30.2	2236	0.822
47	8.10	54.7	2290	0.779
48	5.02	35.7	2258	0.783
49	4.74	29.4	2291	0.821
50	4.58	34.5	2356	0.818
51	4.55	24.3	2494	0.800
52	4.64	40.0	2466	0.826
53	5.20	63.1	2475	0.823
54	6.41	48.2	2377	0.792
55	7.59	32.1	2219	0.815
56	6.95	43.5	2219	0.803

Table S3: Heavy metal concentrations in 56 soil samples (mg/kg)

Sample no	Zn	Cu	Pb	As	Cr	Ni
1	97.03	33.15	40.17	17.28	98.05	29.02
2	215.3	45.92	51.47	24.53	125.91	55.03
3	145.89	44.92	51.11	24.03	116.82	50.01
4	152.71	44.14	57.55	16.33	110.84	46.23
5	194.20	39.29	111.43	19.34	108.32	57.63
6	154.47	37.24	85.48	17.65	90.32	41.11
7	173.38	74.10	51.8	23.50	198.87	68.45
8	106.47	36.32	46.53	14.07	79.32	32.21
9	123.29	44.31	45.06	15.72	177.51	47.60
10	64.11	18.92	29.21	9.74	62.05	29.65
11	142.68	41.89	90.00	21.57	97.41	44.79
12	178.06	131.35	44.36	13.62	134.65	58.76
13	210.72	57.46	72.19	23.14	152.87	57.84
14	166.31	75.46	46.33	26.76	206.45	77.80
15	114.29	58.42	41.69	16.67	110.68	50.91
16	270.65	37.26	55.60	30.92	129.24	60.56
17	336.52	49.28	104.36	23.36	144.63	68.69
18	170.01	39.29	52.26	21.19	83.62	43.22
19	158.26	26.43	66.69	41.02	92.09	33.26
20	278.72	53.54	85.79	23.56	127.6	56.57
21	253.72	67.17	73.05	22.22	124.09	57.27
22	183.81	36.29	67.63	21.21	79.53	42.11
23	258.24	34.99	95.23	22.69	83.72	62.33
24	291.99	64.60	75.05	25.38	129.56	75.62
25	377.22	36.41	115.46	24.65	120.07	56.77
26	240.01	56.38	80.544	44.51	160.37	75.24
27	137.95	40.92	42.94	22.32	108.68	54.01
28	112.29	34.49	47.32	18.56	96.91	42.22
29	160.82	42.19	44.89	25.91	110.75	51.94
30	126.82	36.34	44.31	20.32	109.32	33.78
31	371.78	72.01	74.04	49.49	292.04	97.15
32	468.55	67.52	86.28	37.21	183.31	67.95
33	183.93	34.17	73.23	26.4	103.64	32.03
34	137.07	33.09	41.93	15.41	92.71	43.56
35	166.04	36.19	46.61	35.77	100.15	43.21
36	131.26	39.96	57.34	16.69	97.41	43.04
37	70.96	25.78	21.83	11.28	72.12	52.48
38	123.18	32.79	57.33	17.99	91.13	39.87
39	102.12	26.21	55.11	15.53	83.48	24.89
40	94.49	26.27	42.25	15.74	89.43	25.49

41	170.45	40.00	76.47	17.19	113.72	49.76
42	265.89	44.55	77.80	35.62	99.61	47.60
43	333.98	56.71	65.93	38.05	92.43	74.96
44	154.46	39.63	68.92	32.44	108.0	41.85
45	158.60	24.94	46.65	31.13	78.5	47.71
46	219.40	21.46	36.33	34.78	87.82	35.16
47	125.17	45.43	49.08	35.42	104.24	37.75
48	122.87	37.85	49.58	33.38	105.3	43.24
49	97.11	33.31	40.38	22.53	93.96	32.63
50	201.81	49.87	43.42	37.95	129.94	56.54
51	178.93	43.84	77.48	34.27	114.69	45.33
52	146.92	130.98	19.96	23.76	131.21	63.41
53	168.82	75.23	53.22	24.55	165.73	42.41
54	62.83	88.49	57.94	62.83	224.55	93.10
55	145.37	56.75	45.68	28.95	108.15	56.35
56	175.76	54.17	43.46	37.37	116.1	44.83
Mean	181	47.8	59.3	25.8	119	50.8
SD	81.7	22.0	20.9	10.1	41.2	15.6

Table S4: Pollution load index for metals in soil

**Sample no**	**1**	**2**	**3**	**4**	**5**	**6**	**7**
	0.525	0.812	0.734	0.689	0.836	0.689	0.944
**Sample no**	**8**	**9**	**10**	**11**	**12**	**13**	**14**
	0.527	0.691	0.357	0.743	0.848	0.917	0.97
**Sample no**	**15**	**16**	**17**	**18**	**19**	**20**	**21**
	0.665	1.04	1.05	0.667	0.734	0.979	0.932
**Sample no**	**22**	**23**	**24**	**25**	**26**	**27**	**28**
	0.688	0.835	1.03	1.098	1.38	0.687	0.599
**Sample no**	**29**	**30**	**31**	**32**	**33**	**34**	**35**
	0.729	0.608	1.56	1.42	0.715	0.582	0.724
**Sample no**	**36**	**37**	**38**	**39**	**40**	**41**	**42**
	0.612	0.422	0.607	0.5	0.481	0.743	0.896
**Sample no**	**43**	**44**	**45**	**46**	**47**	**48**	**49**
	1.015	0.768	0.644	0.627	0.71	0.699	0.557
**Sample no**	**50**	**51**	**52**	**53**	**54**	**55**	**56**
	0.86	0.843	0.795	0.854	1.06	0.777	0.803

Table S5: Heavy metal concentration in 56 potato samples (wet weight mg/kg)

Sample no	Zn	Cu	Pb	As	Cr	Ni
1	3.74	1.02	0.048	<0.010	0.035	0.079
2	2.82	0.907	0.011	<0.010	0.029	0.062
3	2.60	0.455	0.010	<0.010	0.024	0.042
4	2.49	0.611	0.024	<0.010	0.026	0.070
5	1.98	0.277	0.010	<0.010	0.022	0.051
6	1.95	0.232	0.011	<0.010	0.021	0.044
7	1.62	0.327	0.010	<0.010	0.025	0.047
8	1.83	0.284	0.010	<0.010	0.042	0.050
9	1.56	0.465	0.022	<0.010	0.027	0.040
10	2.41	0.539	0.043	<0.010	0.028	0.105
11	1.85	0.441	0.032	<0.010	0.016	0.060
12	2.83	1.02	0.016	<0.010	0.024	0.070
13	2.41	0.325	0.034	<0.010	0.044	0.034
14	1.92	0.471	0.018	<0.010	0.034	0.062
15	2.77	0.854	0.016	<0.010	0.016	0.030
16	3.02	0.796	0.024	<0.010	0.019	0.066
17	2.84	0.780	0.018	<0.010	0.021	0.024
18	4.04	1.06	0.038	<0.010	0.066	0.035
19	3.33	0.855	0.053	<0.010	0.022	0.106
20	3.23	0.592	0.034	<0.010	0.018	0.018
21	5.04	0.850	0.022	<0.010	0.018	0.044
22	5.15	0.973	0.021	<0.010	0.023	0.043
23	3.32	0.710	0.021	<0.010	0.023	0.031
24	3.32	0.545	0.024	<0.010	0.020	0.061
25	3.14	0.784	0.034	<0.010	0.024	0.025
26	7.42	1.18	0.094	<0.010	0.026	0.040
27	1.78	0.448	0.025	<0.010	0.019	0.025
28	3.17	0.527	0.024	<0.010	0.011	0.036
29	2.00	0.873	0.018	<0.010	0.016	0.020
30	2.90	0.561	0.022	<0.010	0.010	0.047
31	2.88	0.676	0.050	<0.010	0.023	0.096
32	3.55	0.702	0.095	<0.010	0.529	0.094
33	2.48	0.655	0.022	<0.010	0.021	0.029
34	2.57	0.661	0.032	<0.010	0.016	0.052
35	2.51	0.523	0.017	<0.010	0.107	0.042
36	1.75	0.744	0.019	<0.010	0.015	0.031
37	2.69	0.964	0.038	<0.010	0.021	0.202
38	3.07	1.02	0.029	<0.010	0.036	0.090
39	4.20	0.987	0.024	<0.010	0.016	0.109
40	3.01	0.589	0.025	<0.010	0.013	0.059
41	2.09	0.574	0.025	<0.010	0.030	0.049

42	2.19	0.438	0.017	<0.010	0.017	0.039
43	2.22	0.695	0.015	<0.010	0.016	0.071
44	1.84	0.397	0.035	<0.010	0.023	0.062
45	2.44	0.823	0.036	<0.010	0.022	0.031
46	2.46	0.768	0.018	<0.010	0.021	0.086
47	2.08	0.660	0.054	<0.010	0.017	0.020
48	2.35	0.976	0.035	<0.010	0.044	0.047
49	1.93	0.650	0.016	<0.010	0.543	0.052
50	3.61	1.02	0.033	<0.010	0.659	0.096
51	2.41	0.487	0.014	<0.010	0.539	0.097
52	1.61	0.592	0.017	<0.010	0.492	0.059
53	1.98	0.501	0.012	<0.010	0.018	0.040
54	1.96	0.638	0.011	<0.010	0.015	0.028
55	1.87	0.767	0.044	<0.010	0.026	0.033
56	2.37	0.520	0.030	<0.010	0.021	0.025
Mean	2.73	0.675	0.027	--	0.072	0.055
SD	1.02	0.228	0.018	--	0.153	0.032

Table S6: Bio-concentration factor for potato (dry weight)/soil system

Sample no	Zn	Cu	Pb	As	Cr	Ni
1	0.1985	0.1579	0.0062	<0.001	0.0018	0.0141
2	0.0680	0.1026	0.0011	<0.001	0.0012	0.0059
3	0.0967	0.0549	0.0010	<0.001	0.0011	0.0045
4	0.1005	0.0852	0.0025	<0.001	0.0015	0.0093
5	0.0527	0.0363	0.0010	<0.001	0.0011	0.0046
6	0.0666	0.0328	0.0010	<0.001	0.0012	0.0057
7	0.0518	0.0244	0.0010	<0.001	0.0007	0.0038
8	0.0100	0.0456	0.0010	<0.001	0.0031	0.0090
9	0.0727	0.0602	0.0028	<0.001	0.0010	0.0049
10	0.1923	0.1460	0.0075	<0.001	0.0023	0.0181
11	0.0812	0.0660	0.0022	<0.001	0.0010	0.0084
12	0.0817	0.0397	0.0019	<0.001	0.0010	0.0061
13	0.0621	0.0307	0.0025	<0.001	0.0016	0.0032
14	0.0600	0.0325	0.0021	<0.001	0.0010	0.0041
15	0.1176	0.0709	0.0018	<0.001	0.0010	0.0029
16	0.0589	0.1126	0.0023	<0.001	0.0010	0.0058
17	0.0410	0.0771	0.0010	<0.001	0.0010	0.0017
18	0.1093	0.1245	0.0034	<0.001	0.0036	0.0037
19	0.0212	0.1732	0.0043	<0.001	0.0013	0.0170
20	0.0116	0.0554	0.0020	<0.001	0.0007	0.0016
21	0.0949	0.0604	0.0015	<0.001	0.0010	0.0037
22	0.1451	0.1388	0.0016	<0.001	0.0015	0.0053
23	0.0689	0.1086	0.0012	<0.001	0.0014	0.0026
24	0.0701	0.0521	0.0020	<0.001	0.0010	0.0050
25	0.0408	0.1054	0.0014	<0.001	0.0010	0.0021
26	0.1583	0.1075	0.0060	<0.001	0.0010	0.0028
27	0.0646	0.0549	0.0030	<0.001	0.0010	0.0023
28	0.1433	0.0775	0.0026	<0.001	0.0010	0.0043
29	0.0642	0.1069	0.0021	<0.001	0.0010	0.0020
30	0.1062	0.0717	0.0023	<0.001	0.0010	0.0064
31	0.0422	0.0512	0.0037	<0.001	0.0010	0.0054
32	0.0422	0.0579	0.0062	<0.001	0.0161	0.0077
33	0.0751	0.1073	0.0017	<0.001	0.0012	0.0050
34	0.1114	0.1187	0.0045	<0.001	0.0010	0.0070
35	0.1010	0.0965	0.0024	<0.001	0.0071	0.0064
36	0.0750	0.1047	0.0019	<0.001	0.0010	0.0041
37	0.1959	0.1926	0.0090	<0.001	0.0015	0.0198
38	0.1278	0.1601	0.0026	<0.001	0.0020	0.0116
39	0.2262	0.2071	0.0024	<0.001	0.0011	0.0241
40	0.1706	0.1198	0.0032	<0.001	0.0010	0.0124

41	0.0674	0.0789	0.0018	<0.001	0.0014	0.0054
42	0.0449	0.0537	0.0012	<0.001	0.0010	0.0044
43	0.0334	0.0618	0.0011	<0.001	0.0010	0.0048
44	0.0653	0.0550	0.0028	<0.001	0.0012	0.0081
45	0.0731	0.1570	0.0037	<0.001	0.0013	0.0031
46	0.0629	0.2012	0.0028	<0.001	0.0013	0.0138
47	0.0749	0.0657	0.0050	<0.001	0.0010	0.0024
48	0.0881	0.1190	0.0032	<0.001	0.0019	0.0050
49	0.1113	0.1092	0.0022	<0.001	0.0323	0.0089
50	0.0981	0.1122	0.0041	<0.001	0.0279	0.0093
51	0.0673	0.0554	0.0009	<0.001	0.0234	0.0107
52	0.0626	0.0259	0.0049	<0.001	0.0215	0.0054
53	0.0662	0.0375	0.0013	<0.001	0.0010	0.0053
54	0.1500	0.0347	0.0010	<0.001	0.0010	0.0014
55	0.0697	0.0731	0.0052	<0.001	0.0013	0.0032
56	0.0685	0.0488	0.0035	<0.001	0.0010	0.0028
Mean	0.087	0.088	0.0028	<0.001	0.0034	0.0066
